# Acute physiological responses and performance determinants in Hyrox^©^ – a new running-focused high intensity functional fitness trend

**DOI:** 10.3389/fphys.2025.1519240

**Published:** 2025-03-31

**Authors:** Tom Brandt, Cindy Ebel, Christopher Lebahn, Annette Schmidt

**Affiliations:** Institute of Sports Science, University of the Bundeswehr Munich, Neubiberg, Germany

**Keywords:** fitness racing, HIFT, concurrent training, high intensity interval training, heart rate, maximum oxygen consumption, lactate, relative perceived exertion

## Abstract

**Aims:**

Hyrox^©^ is a fitness modality combining 8 functional exercises with running in a comprehensive competition format. Within this first scientific study on Hyrox^©^, acute physiological responses, relative perceived exertion (RPE), and possible performance determinants were assessed during a simulated Hyrox^©^ competition to derive training recommendations and potential practical applications.

**Methods:**

Eleven recreational Hyrox^©^ athletes [27% women, Hyrox^©^ experience median (interquartile range): 18 (19) months] participated. In a pre-test, height, body composition, hand grip strength (HGS), maximum oxygen consumption (VO_2_max), and volume of resistance and endurance training were assessed. After 48 h rest, a simulated Hyrox^©^ was conducted according to the competition-standards of the “Individual Open Division”. Heart rate (HR) was tracked throughout the Hyrox^©^. Blood lactate (BL) and RPE were recorded at the beginning and after each run and exercise station. Differences between runs and exercise stations for HR, BL, and RPE were analyzed via Wilcoxon signed rank test. Spearman’s rank correlation test was conducted to identify associations between completion times (Hyrox^©^, runs, exercise stations) and participant characteristics. Values are given as median (interquartile range).

**Results:**

Completion time of the Hyrox^©^ was 86.5 (14.5) minutes, whereby runs (51.2 (14.1) minutes) were significantly longer than the exercise stations [32.8 (6.1) minutes] (p = 0.003). Most of the Hyrox^©^ was performed at very hard and hard intensities [79.5 (21)% and 19.6 (20.7)% of maximum HR]. Maximum BL was higher during the exercise stations [8.5 (5.4) mmol/L] compared to the runs (7.7 (4.6) mmol/L) (p = 0.006). Similar results were found for maximum RPE [exercise stations: 18 (2), runs: 16 (2), p = 0.003]. The highest values for HR, BL, and RPE occurred during the last exercise (wall balls). The exercise stations with the heaviest loads were completed the fastest [sled push: 128 (34) seconds, sled pull: 155 (38) seconds]. Faster Hyrox^©^ completion correlated significantly with higher VO_2_max (p = 0.01), greater endurance training volume (p = 0.04), and lower body fat percentage (p = 0.03).

**Conclusion:**

Hyrox^©^ is a HIFT modality with an emphasis on endurance capacity and moderate to low requirements in terms of maximum strength, coordination, and mobility when compared to other forms HIFT. Hyrox^©^ may be suitable for health promotion and tactical population training.

## 1 Introduction

High-intensity interval training (HIIT), strength training with free weights, bodyweight training, and functional fitness training (FFT) have been ongoing trends for years and were among the top 10 trends in the fitness industry in 2023 (Thompson, 2023). Fitness racing such as Hyrox^©^ (Upsolut Sports GmbH, Hamburg, Germany) is a new fitness domain combining elements of these training modalities in a comprehensive competition format (Bergmann, 2024). Similar to CrossFit^©^ (CF) (CrossFit, Inc., Washington, DC, United States) – one of the most popular FFT concepts – Hyrox^©^ also incorporates elements of gymnastics, weightlifting, and endurance sports (Glassman, 2020). In contrast to CF, a Hyrox^©^ competition always consists of the same 8 exercises (ski ergometer, sled push, sled pull, burpee broad jump, rowing ergometer, farmers carry, sandbag lunges, and wall balls) and 8 runs [each 1,000 meters (m)]. Runs and exercises are completed alternately one after the other, whereby the competition always starts with a run. Weights and distances of the exercises are adjusted based on gender, division, and mode (e.g., Individual Open, Individual Pro, Double, Relay), allowing athletes of varying fitness levels to participate. The first Hyrox^©^ competition took place in 2017. Considering that in the 2022/2023 season already 90,000 Hyrox^©^ athletes worldwide competed despite the corona pandemic, a further increase in popularity could be expected (Bergmann, 2024). Nevertheless, Hyrox^©^ is not represented in the scientific literature up to date. Accordingly, little is known about acute physiological responses and performance determinants. However, this information is essential for developing effective training programs and exploring potential application areas of Hyrox^©^ beyond competitive sport. Indeed, previous research on other sports respectively training concepts that appear to be closely related to Hyrox^©^ such as CF, concurrent training (CT), and running may provide some insight in this regard.

Looking at CF, there is a considerable amount of research providing information for coaches, athletes, and health professionals in terms of physiological and psychological demands, performance predictors, injury rates, health benefits, and motivational aspects (Claudino et al., 2018; Dexheimer et al., 2019; Ángel Rodríguez et al., 2022; Brandt et al., 2024; Dominski et al., 2021). Regarding health and fitness, previous research showed that CF improves cardiovascular fitness, body composition, mobility, strength, and back health, while being associated with a low injury risk (1.9 – 3.2 injuries per 1000 hours [h]) (Gianzina and Kassotaki, 2019; Brandt et al., 2024; Hak et al., 2013). In terms of performance, it was demonstrated that a lower body fat percentage, higher total body strength, and maximum oxygen consumption (VO_2_max) correlated with better CF performance (Meier et al., 2023a). However, the investigated CF workouts (e.g., Fran, Cindy, Isabel, Murph) were considerably shorter than a Hyrox^©^ competition (Ahmad et al., 2018; Rios et al., 2024a; Rios et al., 2024b; Carreker and Grosicki, 2020). The single study on physiological demands covering longer bouts of CF was conducted over the course of regular 60-minute [min] CF training sessions (Meier et al., 2023b). This limits the extent to which conclusions could be derived about the physiological demands of Hyrox^©^. Unlike CF, which emphasizes constantly varied functional movements, Hyrox^©^ places a greater focus on running, with a total of 8 kilometers [km] per competition and less exercise variety (Bergmann, 2024; Glassman, 2020).

On endurance exercise such as running, extensive research has been carried out in a large number of different populations with regard to physiological demands, health benefits, or injury patterns. Performance in Olympic endurance events is typically achieved at intensities exceeding 85% of the VO_2_max. Joyner and Coyle concluded that endurance athletes need to be relatively fatigue resistant at intensities that stimulate significant anaerobic metabolism (Joyner and Coyle, 2008). The most important factors especially for running performance therefore include maximal oxygen uptake (VO_2_max), lactate threshold, and running economy (Joyner, 1991). Regarding health benefits, a meta-analysis with a pooled sample of N = 232,149 participants showed that running is associated with a 27%, 30%, and 23% reduced risk of all-cause, cardiovascular, and cancer mortality (Pedisic et al., 2020). Lee et al. stated that, from a public health perspective, running could be the most cost-effective lifestyle medicine, exceeding the importance of risk factors like smoking, obesity, hypertension, or diabetes (Lee et al., 2017). Nevertheless, running is associated with an injury risk of 6.8–59 injuries per 1,000 h whereby history of previous injuries, mileage but also training volume and frequency need to be considered to prevent injury (Saragiotto et al., 2014; Fields et al., 2010). While research on endurance exercise – and in particular running – provides indications regarding the demands and effects of Hyrox^©^, the specific characteristics of Hyrox^©^ (e.g., integration of functional exercises) might lead to divergent findings.

Besides running, Hyrox^©^ includes exercises such as sled pushes (men: 202 kilograms [kg], women: 152 kg) and sled pulls (men: 153 kg, women: 103 kg) where heavy external weights need to be moved (Bergmann, 2024). This suggests that Hyrox^©^ training regimes should also include resistance training to some extent. Notably, resistance training may not only improve competition performance but health (e.g., reducing cardiovascular disease, hypertension, all-cause-mortality, and the offset of age-related declines in strength, power, and muscle mass) (Fyfe et al., 2022). Nevertheless, implementation of additional strength training needs to be done with caution because volume and frequency are critical risk factors for injury (Saragiotto et al., 2014; Fields et al., 2010). Furthermore, previous research in the field of CT showed that combining resistance and endurance training poses a challenge, as the specific adaptations from each type of training may potentially interfere with one another. Therefore, to manage overall fatigue, reduce negative interferences, and avoid injuries, knowing the specific demands of Hyrox^©^ in terms of strength and endurance capacity is essential according to CT research (Methenitis, 2018; Wilson et al., 2012).

However, no scientific studies to date have investigated Hyrox^©^ in this respect, making it difficult to develop evidence-based training programs or identify potential benefits and practical applications beyond competitive sports. Therefore, this study analyzed the acute physiological responses as well as potential performance predictors in Hyrox^©^ athletes during a simulated Hyrox^©^ competition.

## 2 Materials and methods

### 2.1 Trial overview

**FIGURE 1:**
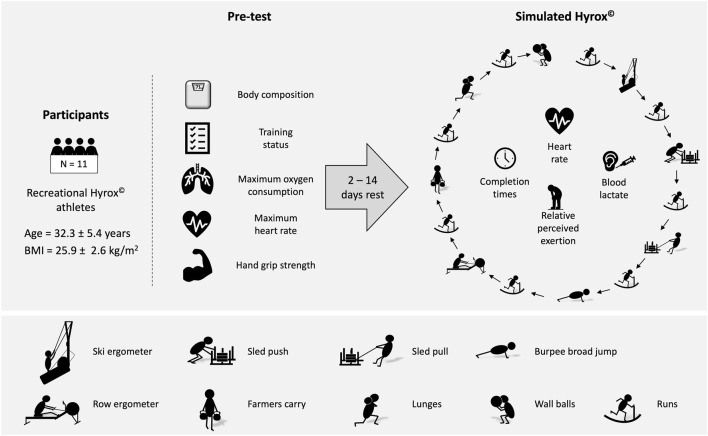
Trial overview.

This study was conducted at the University of the Bundeswehr Munich (UniBw M). Participants attended 2 separate test sessions – a pre-test and a simulated Hyrox^©^ competition. The simulated Hyrox^©^ was performed 2–14 days after the pre-test. The Institutional Ethics Committee of the UniBw M approved the study protocol, ensuring that it conformed to the ethical guidelines of the 1975 Declaration of Helsinki. Informed consent was obtained from all subjects involved in the study (08.02.2024; EK UniBw M 24-05). An overview of the trial is displayed in Figure 1.

### 2.2 Participants

The study involved recreational Hyrox^©^ athletes (N = 11, 27% women, Hyrox^©^ experience: 18 (19) months) who were at least 18 years old and had participated in at least one official Hyrox^©^ competition prior to the study. Exclusion criteria were health issues that would preclude participation in the applied tests (e.g., cardiovascular diseases, respiratory disorders, severe injuries to the musculoskeletal system, osteoporosis, intervertebral disc damage, joint replacements, hypertension). No adverse events occurred during the test sessions. Participant characteristics are displayed in Table 1.

**TABLE 1:** Characteristics of participants.

Parameter	Median	IQR	Min	Max
N	11			
Male (N) Female (N) Diverse	72.7% (8) 27.3% (3) 0%			
Age [years]	33	9	23	43
Body mass [kg]	71.6	20.5	62.4	108.5
BMI [kg/m^2^]	24.2	4.6	23.3	30.6
Body fat [%]	21.3	7.3	10.7	25
Muscle mass [%]	37.7	4.6	34.5	40.8
Hand grip strength [kg]	51.1	10.7	35.1	73.3
VO_2_max [mL/min/kg]	51	10.8	46.6	72
HR_max_ [bpm]	186	24	166	201
Hyrox^©^ experience [months]	18	19	2	48
Endurance training [hours/week]	3	2.5	2	7
Resistance training [hours/week]	4	3	1	7

Abbreviations: BMI, body mass index; bpm, beats per minute; HR_max_, maximum heart rate; kg, kilograms; m, meter; mL, milliliter; min, minute; IQR, interquartile range; VO_2_max, maximum rate of oxygen consumption.

### 2.3 Procedure

#### 2.3.1 Pre-test

The aim of the pre-test was to collect measures of body composition, strength, cardiorespiratory fitness, and training status. Participants were instructed to avoid any intensive physical activity 48 h, alcohol consumption 24 h, and food intake 3 h prior the test. Moreover, they should not drink more than 500 mL of water 30 min before the measurement and empty their bladder. The pre-test comprised a questionnaire, bioelectrical impedance analysis (BIA), hand grip strength (HGS) test, and cardiopulmonary exercise test on a treadmill (CPET).

##### 2.3.1.1 Questionnaire

Participants were asked for their gender and age [years], hours of resistance as well as endurance training per week [h/wk], and Hyrox^©^ experience in months.

##### 2.3.1.2 Body composition and height

Height [cm], weight [kg], and body composition (body fat percentage [%] and muscle mass percentage [%]) were measured in underwear. Height was assessed with a SECA® 213 (seca GmbH and Co. KG, Hamburg, Germany) and body composition with a SECA® mBCA 515 scale (seca GmbH and Co. KG, Hamburg, Germany). According to Bosy-Westphal et al., the SECA® mBCA 515 is a valid and precise tool to estimate body composition (Bosy-Westphal et al., 2017; Bosy-Westphal et al., 2013).

##### 2.3.1.3 Hand grip strength test

HGS was measured with the Baseline® Smedley Digital hand-held dynamometer (Fabrication Enterprises, New York, United States) and given in kg. For the measurement, participants sat upright on a chair with their feet on the ground and a 90° angle in their knee joint. The elbow was flexed at 90° with the fingertips pointed ventrally and the palm facing medially. Starting with the dominant hand, participants were instructed to squeeze the dynamometer as hard as they could for 5 seconds [sec]. Each hand was measured thrice with 30 s of rest between trials, whereby the highest of all 6 measures was used for the analysis. HGS strongly correlates with strength in other muscle groups and can be used as a predictor for total muscle strength (Wind et al., 2010). Furthermore, since Hyrox^©^ is a fitness racing modality including exercises that require athletes to hold and pull external loads during sled pulls and farmers carry, HGS could influence performance.

##### 2.3.1.4 Cardiopulmonary exercise test

The participants performed an incremental exercise test on a h/p/cosmos pulsar® 3p treadmill (h/p/cosmos, Traunstein, Germany). Women started at 6 km/h and men at 7 km/h, both at an incline of 1%. The speed was increased by 1 km/h every 3 min until participants were unable to maintain running pace. As proposed by Rosenerger and Schommer, exhaustion criteria comprised a respiratory exchange ratio >1.1, plateauing of VO_2_ consumption, and a HR reaching or exceeding 100% ± 10% of the age-predicted HR_max_ (Scharhag-Rosenberger and Schommer, 2013). Heart rate (HR) was continuously tracked with a smartLAB hrm W sensor worn at the chest (HMM Diagnostics GmbH, Heddesheim, Germany). To assess cardiopulmonary indices the COSMED Quark CPET (COSMED, Rome, Italy) was used. VO_2_max [mL/min/kg] was determined by averaging the oxygen uptake over the last 30 s at the participant’s peak performance.

#### 2.3.2 Simulated Hyrox^©^ competition

**FIGURE 2:**
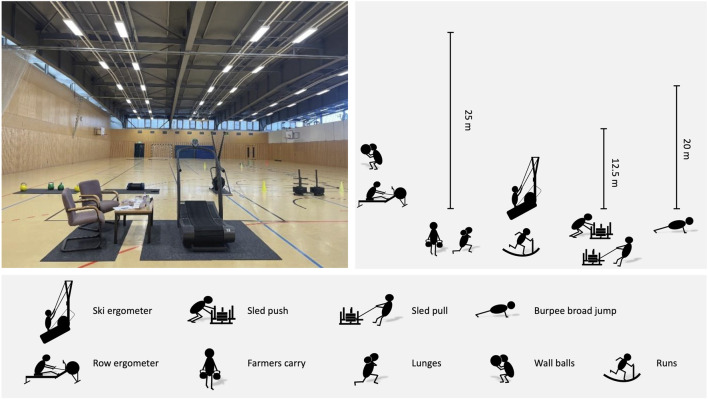
Test setup for the simulated Hyrox^©^ competition.

In the simulated Hyrox^©^ competition test, physiological responses were assessed in order to estimate the demands of a Hyrox^©^ competition. Participants were instructed to avoid any intensive physical activity 48 h prior the test. All participants completed the Hyrox^©^ according to the standards of the “Individual Open Division”. The exercise execution followed the guidelines outlined for Hyrox^©^ competitions (Bergmann, 2024). Each participant completed the test alone in a gym. The 8 runs and exercise stations were performed in alternating order, always starting with a 1 km run on a Woodway Curve treadmill (WOODWAY, Weil am Rhein, Germany). The exercise stations were (1) ski ergometer (1000 m; Concept2, Morrisville, USA), (2) sled push (4 × 12.5m, women: 102 kg, men: 152 kg, including weight of the sled) (3) sled pull (4 × 12.5m, women: 78 kg, men: 103 kg, including weight of the sled), (4) burpee broad jumps (4 × 20 m), (5) rowing ergometer (1000 m; Concept2, Morrisville, USA), (6) farmers carry (8 × 25 m, women: 2 × 16 kg kettle bell, men: 2 × 24 kg kettle bell), (7) sandbag lunges (4 × 25 m, women: 10 kg, men: 20 kg), and (8) wall balls (women: 75 repetitions with 4 kg, men: 100 repetitions with 6 kg). Participants were instructed to complete the Hyrox^©^ as fast as possible. The test setup in the gym is shown in Figure 2.

The completion times were recorded for the full Hyrox^©^, the accumulated runs, the accumulated exercise stations as well as for each run and exercise station. During the test, the participants wore a smartLAB hrm W sensor at the chest (HMM Diagnostics GmbH, Heddesheim, Germany) to track their HR. The maximum heart rate (HR_max_) and average HR (HR_avg_) was determined for the complete Hyrox^©^, as well as for the accumulated runs, accumulated exercise stations, and each individual run and exercise station. The relative HR [%HR_max_] was calculated to the second for the entire Hyrox^©^ to estimate the exercise intensity according to the guidelines of the American College of Sports Medicine (very light, light, moderate, hard, very hard, maximum) (Pollock et al., 1998).

Blood lactate concentration (BL) in millimoles per liter [mmol/L] was measured with a Lactate Scout+ (SensLab GmbH, Leipzig, Germany) before the test, after each run, after each exercise station, and at 3 and 6 min after the completion of the final exercise station. Simultaneously with the lactate measurements, relative perceived exertion (RPE) was assessed using the Borg scale (scale from 6–20 with 6 being the lowest and 20 the highest possible perceived exertion). The scale was presented and explained to the participants beforehand, following the standards for its use in sports medicine as recommended by Löllgen (Borg, 1998; Löllgen, 2004). The maximum BL (BL_max_), maximum RPE (RPE_max_), average BL (BL_avg_), and average RPE (RPE_avg_) was determined for the complete Hyrox^©^, as well as for the accumulated runs, and accumulated exercise stations.

### 2.4 Statistical analysis

The statistical analysis was conducted with Microsoft Excel (Microsoft, Redmond, United States) and SPSS 29® (IBM SPSS, Armonk, NY, United States). Normal distribution was analyzed via Kolmogorov-Smirnov test and Q-Q-plots. Values are given as median (interquartile range). Differences between the runs and workout stations in terms of physiological responses and RPE were analyzed via Wilcoxon signed rank test. The effect size r was calculated based on the z-value and the sample size. Spearman’s rank correlation test was performed to identify associations between the completion times (complete Hyrox^©^, runs, exercise stations) and participant characteristics (e.g., measures of body composition, strength, and endurance). Spearman’s rho was reported as the correlation coefficient. Statistical significance was set at p ≤ 0.05.

## 3 Results

**FIGURE 3:**
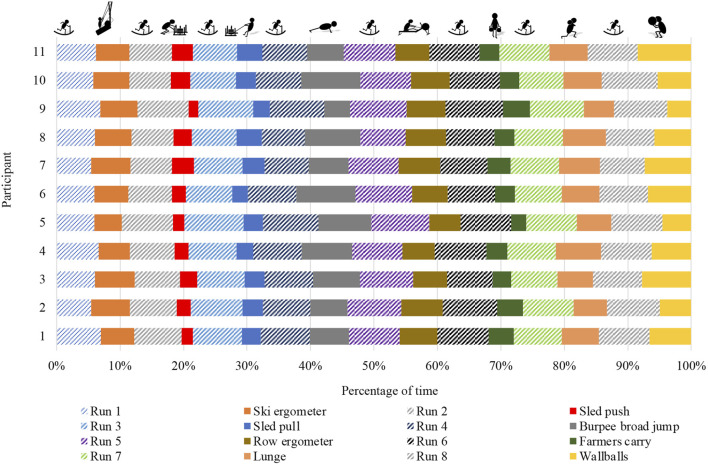
Distribution of the time spent completing the exercise stations and runs during the simulated Hyrox^©^ competition.

Participants completed the Hyrox^©^ in 86.5 (14.5) min, whereby the time spent running [51.2 (14.1) min] was significantly longer than the time to complete the exercise stations [32.8 (6.1) min] (p = 0.003, r = 0.88). The percentage share of the specific exercise stations and runs in the total time to completion is displayed in Figure 3. The shortest median times to completion occurred for sled push [2.1 (0.6) min], sled pull [2.6 (0.6) min], and farmers carry [2.7 (0.7 min]. Run 5 [7.4 (1.6) min], Run 8 [6.8 (1.3) min], and Run 6 [6.8 (1.9) min] took participants the longest.

**FIGURE 4:**
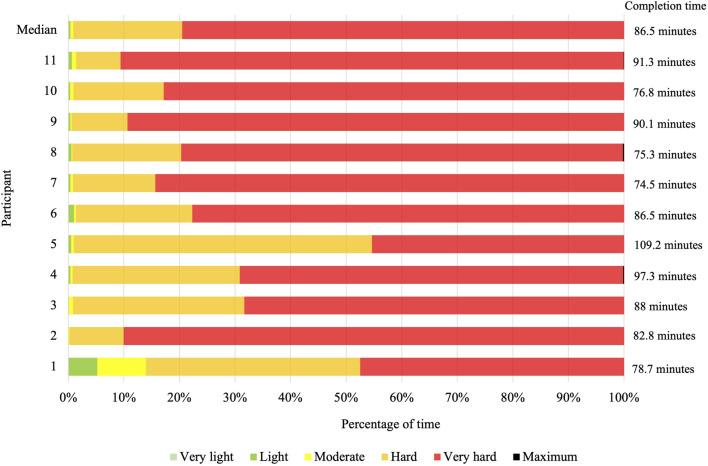
Percentage of time spent in different intensity ranges during the simulated Hyrox^©^competition. Note: Intensity ranges were determined based on the percentage of participants’ maximum heart rate (very light: <35%, light: 35%–55%, moderate: 55%–70%, hard: 70%–90%, very hard: 90%–100%, maximum = 100%).

During the Hyrox, participants achieved a HR_max_ of 185 (26) bpm, with significantly higher values for the exercise stations [185 (26) bpm] compared to the runs [180 (26) bpm] (p = 0.04, r = 62). Similarly, the HR_avg_ in the full Hyrox^©^ reached 170.9 (21.2) bpm while participants completed the exercise stations at a higher HR_avg_ (173.7 (19.5) bpm) compared to the runs [168.9 (21.9) bpm] (p = 0.03, r = 64). Most of the time, participants performed at very hard [79.5 (21) %] and hard [19.6 (20.7) %] intensities. The distribution of the intensity ranges is shown in Figure 4.

Physiologically, the hard to very hard intensity was also reflected in the values for BL_max_ [8.5 (5.4) mmol/L] during the Hyrox^©^. Again, BL_max_ was significantly higher after the exercise stations [8.5 (5.4) mmol/L] compared to the runs [7.7 (4.6) mmol/L] (p = 0.006, r = 83). Looking at BL_avg_ during the Hyrox^©^ [6.3 (3.8) mmol/L], the values after the workouts [6.6 (3.7) mmol/L] were higher in comparison to the runs [6.2 (3.9) mmol/L] (p = 0.003, r = 0.88).

The RPE_max_ score during the Hyrox^©^ reached 18 (2), whereby the participants rated the exercise stations [18 (2)] as significantly more strenuous than the runs [16 (2)] (p = 0.003, r = 0.91). Likewise, participants had a RPE_avg_ of 14.1 (1.5) in the Hyrox^©^, with significantly higher values during the exercise stations [14.4 (1.9)] compared to the runs [13.4 (1.3)] (p = 0.003, r = 0.89). Values for physiological responses and RPE are given in Table 2.

**TABLE 2:** Physiological responses and relative perceived exertion during the simulated Hyrox^©^ competition.

	Full Hyrox^©^	Runs	Exercise stations	r	p
Completion time [min]	86.5 (14.5)	51.2 (14.1)	32.8 (6.1)	0.88	**0.003**
HR_max_ [bpm]	185 (26)	180 (25)	185 (26)	0.62	**0.04**
HR_avg_ [bpm]	170.9 (21.2)	168.9 (21.9)	173.7 (19.5)	0.64	**0.03**
BL_max_ [mmol/L]	8.5 (5.4)	7.7 (4.6)	8.5 (5.4)	0.83	**0.006**
BL_avg_ [mmol/L]	6.3 (3.8)	6.2 (3.9)	6.6 (3.7)	0.88	**0.003**
RPE_max_ score	18 (2)	16 (2)	18 (2)	0.91	**0.003**
RPE_avg_ score	14.1 (1.5)	13.4 (1.3)	14.4 (1.9)	0.89	**0.003**

Note: Values are expressed as median (interquartile range). Effect sizes and significance levels were calculated for differences between runs and Exercise stations. The significance level was set at p < .05. The effect size is given as Pearson´s r. Significant results are displayed in bold.

Abbreviations: BL_avg_, average blood lactate; BLmax, maximum blood lactate HR_avg_, average heart rate; HR_max_, maximum heart rate, bpm = beats per minute, max = maximum, min = minimum, RPE_avg_, average relative perceived exertion; RPE_max_, maximum relative perceived exertion.

**FIGURE 5:**
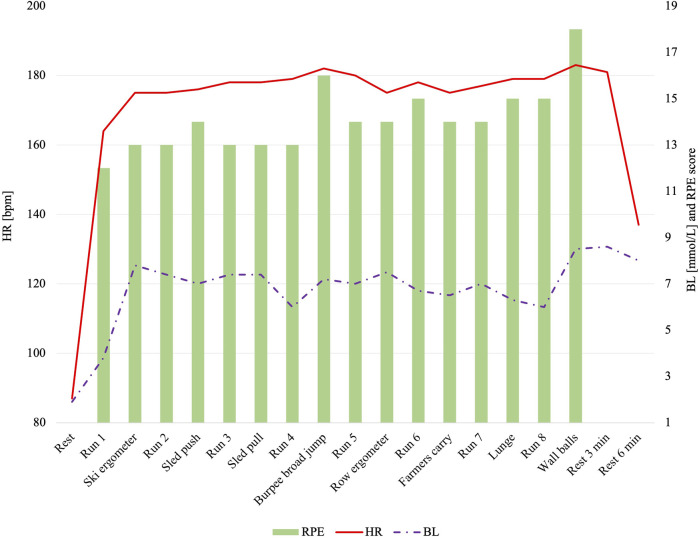
Heart rate, blood lactate, and relative perceived exertion for each exercise station and run during the simulated Hyrox^©^competition. Abbreviations: BL, blood lactate; bpm, beats per minute; HR, maximum heart rate; RPE, relative perceived exertion, mmol/L, millimoles per liter.

The highest values for HR_max_ [183 (21) bpm], BL [8.5 (4.9)], and RPE [18 (1)] were seen in the last exercise station, the wall balls. The course of the HR as well as BL and RPE values are displayed for the Hyrox^©^ in Figure 5.

Correlation analyses showed multiple associations between the participant characteristics and performance in the Hyrox^©^. The strongest correlation was found for VO_2_max (ρ = - 0.71, p = 0.01), followed by endurance training volume (ρ = - 0.68, p = 0.04), and body fat percentage (ρ = 0.67, p = 0.03). Independent analyses of performance during runs and workouts with participants characteristics revealed correlations only for the runs, but not the exercise stations. Strong correlations were seen for VO_2_max (ρ = - 0.73, p = 0.01) and body fat percentage (ρ = 0.68, p = 0.02). Results of the correlations analyses are shown in Table 3.

**TABLE 3:** Correlations between Hyrox^©^ performance and participant characteristics.

	Full Hyrox		Runs		Exercise stations	
	Spearman’s ρ	p	Spearman’s ρ	p	Spearman’s ρ	p
VO_2_max	−0.71	**0.01**	0.73	**0.01**	−0.11	0.74
Body mass	0.02	0.96	−0.11	0.75	0.48	0.14
BMI	−0.07	0.83	−0.16	0.65	0.28	0.4
Body fat percentage	0.67	**0.03**	0.68	**0.02**	0.12	0.72
Muscle mass percentage	−0.03	0.94	−0.07	0.83	0.21	0.53
Hand grip strength	0.09	0.79	−0.03	0.94	0.53	0.09
Endurance training volume	−0.68	**0.04**	−0.63	0.07	−0.43	0.25
Resistance training volume	0.34	0.31	0.29	0.39	0.35	0.3

Note: The time to completion for the complete Hyrox^©^, the accumulated runs, and the accumulated exercise stations were tested for correlations with participant characteristics. The significance level was set at p < 0.05. The correlation coefficient is given as Spearman´s ρ. Significant results are displayed in bold.

Abbreviations: BMI, body mass index; VO_2_max, maximum rate of oxygen consumption.

All participants had the technical proficiency and mobility to perform the exercises according to the Hyrox^©^ movement standards. They further had sufficient strength to execute all exercise station with the competition-standard loads.

## 4 Discussion

### 4.1 Classification and physiological demands of Hyrox^©^

The observed physiological responses and RPE clearly illustrated the high-intensity nature of Hyrox^©^ (Pollock et al., 1998). Given that Hyrox^©^ comprises multi-joint, compound movements performed at high intensities, with loads reaching up to 152 kg for women and 202 kg for men, Hyrox^©^ can be classified as a HIFT modality (Feito et al., 2018). Since more than 60% of the total completion time was spent running, Hyrox^©^ should be further specified as a running-focused form of HIFT.

Based on the overall duration [86.5 (14.5) min] in conjunction with HR and BL values, it is to assume that participants had to rely on both aerobic as well as anaerobic metabolic pathways to complete the Hyrox^©^. Compared to the runs, the exercise stations were significantly more intense indicating higher energy demands per time. In this regard, it is to note that the exercise stations with the heaviest external loads (sled push and sled pull) were completed the fastest. Median completion times of these movements still ranged between 2–3 min indicating that Hyrox^©^ requires athletes to exert a considerable amount of force over prolonged periods of time with anaerobic glycolysis being a key contributor to energy production during these phases. The importance of anaerobic energy production was also confirmed for other HIFT domains such as CF. However, Fernandez et al. and Forte et al. reported considerably higher BL values for CF workouts (mean ± standard deviation: up to 15.8 ± 4.9 mmol/L) (Fernández et al., 2015; Forte et al., 2022). This is in line with recent findings of Sousa Neto et al. who investigated physiological responses after the CF benchmark workout “Karen” (150 repetitions of wall balls with a 9 kg ball as fast as possible) and found significant increases of BL reaching even higher values 17.5 ± 3 mmol/L) (Sousa Neto et al., 2022). Nevertheless, most of the investigated CrossFit^©^ workouts were shorter than 20 min (Fernández et al., 2015; Forte et al., 2022; Sousa Neto et al., 2022). One exception might be the CF workout Murph, in which physiological responses similar to Hyrox^©^ were observed (HR_max_ = 185.6 ± 7.6 bpm, HR_avg_ = 168.8 ± 6.4 bpm, and BL = 10 ± 3 mmol/L). However, Murph was completed substantially faster (43.4 ± 4.6 min) and classified by shorter running distance (2 miles) and time (17.6 ± 2 min) leading to divergent demands for the athletes (Carreker and Grosicki, 2020).

On the other hand, unlike HIFT modalities such as CF where athletes are prepared for “the unknown and unknowable,” exercises but also the loads being moved in Hyrox^©^ competitions are fixed (Bergmann, 2024; Glassman, 2020). This suggests that once strength and power exceed a certain level, benefits of further improvements in these parameters might diminish. Contrastingly, the importance of muscular endurance (e.g., utilizing, tolerating, and clearing lactate) could become more prominent in order to sustain the repeated high-intensity efforts. In this context, it is worth mentioning that the various movements performed in Hyrox^©^ involve the upper as well as lower body musculature (Bergmann, 2024). It is therefore to assume, that both the transition between fatiguing upper-body-dominant as well as lower-body-dominant movements and running inhibits running performance and requires specific adaptions (e.g., redistribution of blood to the legs after movements involving large amounts of upper body musculature; running after high lactate accumulation in the legs due to lower-body-dominant movements). Running under such circumstances could have negatively affected running technique respectively economy which is highly relevant to achieve high running speeds. This could explain the relatively low running pace during the Hyrox^©^ compared to that observed in recreational half-marathon runners (Ogueta-Alday et al., 2018). Therefore, economizing the transition between different metabolic demands as well as maintaining efficient technical execution under fatigue seem to be important aspects for Hyrox^©^ athletes. This is in line with the findings of Peeling et al., who showed that swimming at too high an intensity has detrimental effects on subsequent cycling performance as well as overall performance in triathletes (Peeling et al., 2005). Similarly, Ribiero et al. reported lower overall performance in the CF workout “Fight Gone Bad” when participants completed it in all-out pacing compared to a controlled-split pacing strategy (Ribeiro et al., 2024).

In comparison to endurance sports (e.g., running, cycling, rowing, swimming), Hyrox^©^ athletes need to excel in a greater variety of movement patterns and components of physical fitness (Bergmann, 2024). However, compared to CF, the degree of variation in terms of movements, distances, and loads, the technical demands appear to be relatively low (Bergmann, 2024; Glassman, 2020). This was also displayed by the fact that all participants were able to perform the movements according to Hyrox^©^ competition standards without any scaling. It can therefore be assumed that as performance level and technical proficiency increase, the optimization of metabolic adaptations and proper pacing becomes increasingly important for Hyrox^©^ athletes.

### 4.2 Performance determinants

The correlation analyses were in line with the assumptions drawn from the observed physiological responses and RPE. Strong correlations with VO_2_max suggested that aerobic capacity could be an important performance determinant for Hyrox^©^ athletes. This was also evident in previous research investigating half-marathon runners as well as CF athletes (Meier et al., 2023a; Nikolaidis and Knechtle, 2023). The proximity to CF and running disciplines was further reflected in the strong correlations between the completion times of the Hyrox^©^ and runs with body fat percentage (Meier et al., 2023a; Nikolaidis and Knechtle, 2023). However, in contrast to CF, where measures of muscular strength were found to be important performance determinants, neither HGS nor muscle mass percentage correlated with performance in the present study (Meier et al., 2023a). This was also reflected in the lack of correlation between resistance training and completion times. On the other hand, endurance training volume was strongly associated with completion times of the Hyrox^©^ as well as the runs. However, more information regarding training parameters (e.g., volume, intensity, exercise selection, training history) would be required to better estimate the relevance of resistance and endurance training. Furthermore, only maximum HGS but no direct assessment of lower body strength or strength endurance was conducted.

### 4.3 Training recommendations

When training for a Hyrox^©^, a broad training strategy needs to be applied. According to the observations in the present study, endurance training should to be emphasized to perform a Hyrox^©^ at hard to very hard intensities. Hyrox^©^ training programs should therefore include endurance activities at moderate intensities as well as forms of HIIT to improve aerobic as well as anaerobic capacity. Since athletes spent the majority of time running, a substantial amount of training volume has to be dedicated to running-based training sessions. However, excessive running mileage has been shown to increase the injury risk and should be treated with caution (Fields et al., 2010). A strategy to avoid running-related overuse or injury would be to replace or combine running with other Hyrox^©^ specific endurance activities such as rowing or skiing (Bergmann, 2024). Infact, combining running with other Hyrox^©^ movements should be an integral part of Hyrox^©^ training to improve running economy in a pre-fatigued state and the ability to transition between different metabolic demands.

Although strength appeared to be less important, athletes still need to be able to move external weights according to the competition standards. Previous research on CT indicated that endurance and resistance training may interfere with each other, affecting overall adaptations. To minimize interference, Methenitis recommended separating resistance and endurance training into distinct sessions. When endurance and resistance training have to be done in the same session, the most important aspect should be trained first. In case of athletes that substantially lack maximum strength, power, or muscular endurance to handle competition-standard loads, a training cycle focusing on resistance training could be utilized. For optimal resistance training adaptations, a ratio of 2:1 or 3:1 (resistance: endurance training) in terms of training frequency is recommended. On the other hand, optimal endurance training adaptions should be achieved with a ratio of 1:1 or 1:2 (resistance: endurance training) (Methenitis, 2018). Due to the high-intensity nature of Hyrox^©^, an efficient training method for Hyrox^©^ athletes should be HIIT as it effectively improves measures of endurance such as VO_2_max and leads to less interference effects compared with high-volume, moderate-intensity endurance training (Methenitis, 2018; Mallol et al., 2019). Furthermore, by incorporating Hyrox^©^ exercises and running within a HIIT-style endurance protocol, multiple training objectives could be achieved within a single session and thus minimize overall fatigue. Such training protocols would closely mirror the demands of a Hyrox^©^ competition, possibly enabling athletes to refine transitions between exercises, enhance movement efficiency, and adapt to the specific metabolic demands of Hyrox^©^. While findings of this study allowed for several general training recommendations, assessing individual capabilities and circumstances remains detrimental to design an effective, customized training program.

### 4.4 Perspectives for practice and research

#### 4.4.1 Health promotion

As a form of HIFT, Hyrox^©^ shows a significant overlap with the top recent fitness trends such as HIIT, FFT, and bodyweight training (Thompson, 2023). It is therefore to assume that its popularity will further increase. Besides the competitive execution of Hyrox^©^, it could also be attractive for health-oriented individuals. As outlined above, Hyrox^©^ training should include endurance training at moderate and high intensities as well as resistance training including all major muscle groups. Hyrox^©^ training therefore appears as an efficient way to meet the guidelines of the World Health Organization (WHO) (Bull et al., 2020). The effectiveness of HIFT in terms of fitness and health (e.g., body composition, musculoskeletal, and cardiorespiratory fitness) has already been shown in previous studies (Claudino et al., 2018; Brandt et al., 2024; Bycura et al., 2017). Additionally, physical activity interventions based on Hyrox^©^, could benefit from positive motivational aspects that were found in other forms of HIFT such as CF indicating great potential for behavioral change and maintenance (Brandt et al., 2024; Bycura et al., 2017). The lower number and complexity of exercises compared to CF could further reduce barriers for participation in diverse populations with reduced physical capabilities (e.g., overweight and obese individuals, older adults, and those with physical impairments) (Winter and Gutman, 2022). Nevertheless, Hyrox^©^ still provides considerably more variation than other endurance activities (e.g., running, cycling, swimming) which has been reported to facilitate exercise participation among fitness practitioners (Winter and Gutman, 2022). Due to its similarity to CF, it is to assume that Hyrox^©^ participants also benefit from intrinsic motives such as enjoyment, challenge, and affiliation that support long-term adherence (Fisher et al., 2017). Furthermore, Hyrox^©^ offers different divisions with scaled competition standards, allowing participants of varying fitness levels and age groups to compete with each other (Bergmann, 2024). This does not only increase the accessibility of Hyrox^©^ for different populations, but might additionally results in levels of interest and enjoyment typically seen in sports (Frederick and Ryan, 1993).

#### 4.4.2 Tactical populations

Another potential application area could be the training of tactical populations including soldiers, firefighters, and law enforcement personnel. These groups have to be prepared for a high variety of physical tasks such as dismounted patrols, casualty carry, or intensive combat situations while carrying heavy equipment. Hyrox^©^ could address the need for versatile fitness adaptions (e.g., strength, power, aerobic, and anaerobic capacity) in tactical populations and additionally teach movement patterns such as lifting, pulling, pushing, carrying, and throwing external loads that are common in mission tasks (Bergmann, 2024). Previous studies have already demonstrated the potential of HIFT for tactical populations. Haddock et al. concluded that HIFT modalities (e.g., CF, SEALFIT, US Marine Corps’ High Intensity Tactical Training) provide several benefits such as improved conditioning and strength, general physical preparedness for unpredictable physical demands, less injury potential, low equipment costs, high scalability, and 25%–80% lower training volume compared to conventional military fitness training (Haddock et al., 2016).

#### 4.4.3 Implications for future research

While the present study provided a valuable foundation regarding the characteristics of Hyrox^©^, further research that builds on these results and addresses the limitations identified in this study is needed to gain a comprehensive understanding of this novel HIFT modality. Firstly, it is important to note that all but one participant in the present study demonstrated very good to excellent cardiorespiratory fitness levels based on their VO_2_max values (exception was one male participant showing good cardiorespiratory fitness) (Heyward, 2006). In terms of strength, again 8 participants showed above-average HGS for their age-group whereby only 3 male participants ranged slightly below-average (93.2%–99.6% of reference values) (Ewald and Kohler, 1991). This needs to be considered since physiological differences between athletes differing in experience or fitness level could affect performance and physiological responses (Mangine et al., 2020; Lorenz et al., 2013). Consequently, together with the small sample size, the generalizability of the present findings remains limited. To establish a more nuanced understanding of the characteristics of Hyrox^©^ across different fitness levels, future studies could include a larger, more heterogenous sample. An alternative approach might be to continue working with small sample sizes but homogeneous groups (e.g., only elite men or women), allowing for more distinct conclusions regarding the specific cohort, as was done by Martínez-Gómez et al. and Sauvé et al. for elite CF athletes (Sauvé et al., 2024; Martínez-Gómez et al., 2020). Furthermore, investigations should be extended to other Hyrox^©^ divisions besides the “Individual Open” (e.g., Individual Pro, Doubles, Relay).

Further limiting factors of the present study were that each participant performed the Hyrox^©^ alone (no competitors or spectators), runs did not take place on a track, and the sled used differed from the official competition sled. Especially running on a competition-standard track could lead to different results. For instance, Peserico and Machado found lower running velocity and HR_avg_ but higher HR_max_ when participants ran 60 min on a treadmill compared to a track. Additionally, the authors reported differences in pacing strategies between the track and treadmill condition (Peserico and Machado, 2014). Similar results were confirmed in previous studies investigating shorter distances (Nummela et al., 2007; Morin and Sève, 2011). In future studies, efforts should be made to replicate competition conditions more accurately or measure athletes during official competitions. To enhance the understanding of potential performance determinants, it is also recommended to expand the test battery with additional measurements. An effective method could be the determination of a total athleticism score, as recently proposed by Tibana et al. in the context of CF. The authors calculated the score based on body fat percentage, VO_2_max, muscle power, and muscle endurance providing a holistic measure for overall athleticism (Tibana et al., 2024). A similar tool could be developed and applied in Hyrox^©^, incorporating Hyrox-specific movements and accessible tests (e.g., the critical speed test). Lastly, the potential effectiveness of Hyrox^©^ in terms of health-promotion and tactical training has not been confirmed yet and must be investigated in future studies. In this context, future research should focus not only on fitness and health adaptions but also on motivational aspects, the potential for behavioral change and maintenance, training time and volume, costs, equipment requirements, injury prevalence as well as the applicability across various settings (e.g., commercial gyms, workplace health promotion, military bases, and during deployments abroad).

## 5 Conclusion

Hyrox^©^ is an endurance-focused form of HIFT that combines running with 8 different functional movements. Performing a Hyrox^©^ required athletes to engage both aerobic as well as anaerobic metabolic pathways and to transition between varying metabolic demands depending on the executed movement. Participants spent the majority of the Hyrox^©^ running whereas the intensity according to HR, BL, and RPE values was higher during the exercise stations. Although endurance capacity respectively training appeared to be of greater importance for performance than measures of strength, individual weaknesses should be considered to design effective and sustainable training programs. In this regard, it is suggested to apply CT training recommendations (Methenitis, 2018). Despite the limitations of this first Hyrox^©^ study and the need for further investigations, Hyrox^©^ has emerged as a form of HIFT likely to gain further popularity and offering practical applications beyond its competitive nature, including health promotion and tactical population training.

## Data Availability

The datasets presented in this article are not readily available because The data that support the findings of this study are available from the corresponding author upon reasonable request. Requests to access the datasets should be directed to tom.brandt@unibw.de.
